# Distributed and hierarchical neural encoding of multidimensional biological motion attributes in the human brain

**DOI:** 10.1093/cercor/bhad136

**Published:** 2023-04-28

**Authors:** Ruidi Wang, Xiqian Lu, Yi Jiang

**Affiliations:** State Key Laboratory of Brain and Cognitive Science, CAS Center for Excellence in Brain Science and Intelligence Technology, Institute of Psychology, Chinese Academy of Sciences, 16 Lincui Road, Beijing 100101, China; Department of Psychology, University of Chinese Academy of Sciences, 19A Yuquan Road, Beijing 100049, China; Chinese Institute for Brain Research, 26 Science Park Road, Beijing 102206, China; State Key Laboratory of Brain and Cognitive Science, CAS Center for Excellence in Brain Science and Intelligence Technology, Institute of Psychology, Chinese Academy of Sciences, 16 Lincui Road, Beijing 100101, China; Department of Psychology, University of Chinese Academy of Sciences, 19A Yuquan Road, Beijing 100049, China; Chinese Institute for Brain Research, 26 Science Park Road, Beijing 102206, China; State Key Laboratory of Brain and Cognitive Science, CAS Center for Excellence in Brain Science and Intelligence Technology, Institute of Psychology, Chinese Academy of Sciences, 16 Lincui Road, Beijing 100101, China; Department of Psychology, University of Chinese Academy of Sciences, 19A Yuquan Road, Beijing 100049, China; Chinese Institute for Brain Research, 26 Science Park Road, Beijing 102206, China

**Keywords:** biological motion, emotional state, facing direction, fMRI, gender

## Abstract

The human visual system can efficiently extract distinct physical, biological, and social attributes (e.g. facing direction, gender, and emotional state) from biological motion (BM), but how these attributes are encoded in the brain remains largely unknown. In the current study, we used functional magnetic resonance imaging to investigate this issue when participants viewed multidimensional BM stimuli. Using multiple regression representational similarity analysis, we identified distributed brain areas, respectively, related to the processing of facing direction, gender, and emotional state conveyed by BM. These brain areas are governed by a hierarchical structure in which the respective neural encoding of facing direction, gender, and emotional state is modulated by each other in descending order. We further revealed that a portion of the brain areas identified in representational similarity analysis was specific to the neural encoding of each attribute and correlated with the corresponding behavioral results. These findings unravel the brain networks for encoding BM attributes in consideration of their interactions, and highlight that the processing of multidimensional BM attributes is recurrently interactive.

## Introduction

Precisely perceiving and understanding multidimensional information from other people’s movements play a vital role in our survival and social interaction (Yovel and O’Toole 2016). Bodily movements contain plentiful and hierarchical attributes, including physical attributes like direction and speed, biological attributes like gender and age, and social attributes like affect and intention. Since the point-light biological motion (BM), which isolates human body movements, was introduced by Johansson (1973), a large body of research has revealed that the human visual system can efficiently extract and process distinct physical, biological, and social attributes from BM, such as facing direction (Saunders et al. 2010; Wang et al. 2020), gender (Kozlowski and Cutting 1977; van der Zwan et al. 2009), and emotional state (Atkinson et al. 2004; Clarke et al. 2005; Parkinson et al. 2017). Despite that the ability to recognize and evaluate different BM attributes has been well demonstrated at the behavioral level, it remains largely unknown how the brain simultaneously encodes distinct attributes from BM.

Previous studies have identified a large brain network dedicated to BM processing, involving the posterior superior temporal sulcus (pSTS), the middle temporal visual complex (MT), the fusiform gyrus (FG), and portions of the frontal and parietal cortex (Bonda et al. 1996; Grossman and Blake 2002; Saygin 2004, 2007; Peuskens et al. 2005; Peelen et al. 2006; Jastorff and Orban 2009; Grosbras et al. 2012; Thompson and Parasuraman 2012; van Kemenade et al. 2012; Yovel and O’Toole 2016; Hirai and Senju 2020; Pitcher and Ungerleider 2021). Among this distributed network, certain brain areas are found to be crucial for the processing of some particular attributes of BM. Facing direction perception is supported by FG (Michels et al. 2009), primary somatosensory cortex, and inferior frontal gyrus (IFG; De Lussanet et al. 2008). Emotional state perception network mainly includes pSTS, FG, temporoparietal junction, and superior frontal gyrus (SFG; Alaerts et al. 2014; Atkinson et al. 2012; Goldberg et al. 2015; Jastorff et al. 2016, 2015; Poyo Solanas et al. 2020). On the other hand, the perceptual and neural representations of distinct BM attributes may not be independent of each other, considering the mutidimensional nature of BM. Indeed, a few behavioral studies have shown that the perception of one BM attribute can be significantly modulated by the processing of the other attribute(s). For instance, emotional states conveyed by BM significantly affect its gender categorization such that angry BMs are overwhelmingly judged to be men while sad BMs are judged to be women (Johnson et al. 2011). Gender information also affects the direction discrimination of BM, with the direction discrimination being worse when the BM is depicted as a male than a female (Yang et al. 2014). Based on these observations, it is well expected that there exists a hierarchical neural mechanism for processing multidimensional BM attributes in the brain. In the present study, we investigated how the brain encodes multidimensional BM attributes and in what order these processes are organized. In particular, whether different attributes are extracted and represented in an independent or feedforward manner (e.g. from physical to biological and social attributes), or the processing of multidimensional attributes is accomplished through a recurrently interactive means.

To investigate this issue, we used functional magnetic resonance imaging (fMRI) to measure the hemodynamic response in the brain when participants viewed the multidimensional BM stimuli. Point-light walkers were employed as stimuli that preserve facing direction, gender, and emotional state attributes. We used representational similarity analysis (RSA, Kriegeskorte et al. 2008) because it has the unique advantage of isolating target attributes from the multidimensional BM stimuli in the brain. We first performed a multiple regression RSA to, respectively, identify the brain network in response to each BM attribute. Then we introduced the scrambled representational dissimilarity matrices (RDMs) in RSA to further investigate how the neural representations of BM attributes were influenced by each other. We also used multivoxel pattern analysis (MVPA) methods (Edelman et al. 1998; Kriegeskorte et al. 2008) to explore whether the brain areas identified by RSA were specific to the neural encoding of BM attributes. Lastly, we analyzed the correlations between behavioral results and neural responses by performing an RSA with behavior RDMs. Our study unraveled a distributed and hierarchical neural network dedicated to the neural encoding of multidimensional BM attributes.

## Materials and methods

### Participants

In total, 24 neurologically normal volunteers (12 female, aged 21–30 years) with normal or corrected-to-normal vision participated in the study. Two participants were excluded due to excessive head motion (˃2 mm of maximal translation in any direction of x, y, or z or 2° of any angular motion throughout the scan), and two additional participants were excluded due to their responses in behavioral experiment (see below for details). Participants gave written informed consent prior to participation in the study. The experimental procedures were approved by the institutional review board of the Institute of Psychology, Chinese Academy of Sciences.

### Experimental design and stimuli

#### Stimuli

**Fig. 1:**
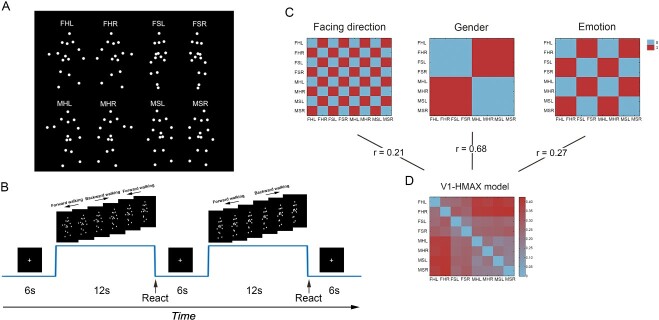
Stimuli, task, and RDMs. (A) Illustration of a single frame of the eight BM sequences. F: Female; M: Male; H: Happy; S: Sad; L: Left; R: Right. (B) Schematic representation of the fMRI experiment. (C) Theoretical RDMs for each attribute. 0 means within category and 1 means between categories. (D) V1 RDM calculated by the HMAX model.

Stimuli were point-light BM sequences based on the motion capture data computed from 50 men and 50 women (https://www.biomotionlab.ca/html5-bml-walker/, Troje 2002). Each stimulus was represented by a set of 15 dots and subtended a visual angle of 6.9^o^ × 3.9^o^. We systematically manipulated the three attributes of the stimuli, which were facing direction (left or right), gender (female or male), and emotional state (happy or sad) (Fig. 1A), leading to eight BM sequences. For facing direction, the point-light walkers were represented in 45^o^ or 135^o^ view. For gender and emotional state, corresponding parameters of the point-light walkers were selected in ±6 standard deviation increments from an average walker (stimulus 0; for details of the metric and associated assumptions, Troje 2008, 2002). The stimuli were presented as white dots against a black background, lasting 1 s in each walking cycle. Stimulus presentation and response collection were controlled by Matlab (MathWorks, Inc.) together with the Psychtoolbox extensions (Brainard 1997).

#### Behavioral experiment

We employed a behavioral experiment prior to the fMRI experiment to test the participants’ abilities for discriminating each attribute of BM. On each trial, one of the BM sequences was presented at the upper center of the screen, and a question with options was presented at the lower center of the screen. We used the semantic differential scale, in which an adjective was paired with its antonym, and the two adjectives are assigned numbers on a scale from 1 to 7 (Osgood 1964). Participants were asked to estimate where an attribute of the stimulus is placed on the scale. For example, in a trial, the question was “what is the gender of the person”, and the option “pretty confident it is a woman” was given 1, and “pretty confident it is a man” was given 7. Each of the eight BM sequences was estimated by its facing direction, gender, and emotional state four times, leading to a total of 96 trials. Behavioral results were presented in Supplementary Material Table S1. The participants who reached the following two exclusion criteria were excluded and did not participate in the fMRI experiment: (i) the behavioral results exceeded three-sigma limits in at least one attribute; (ii) reporting “4—not sure” in ˃50% trials for at least one attribute. According to the exclusion criteria, two participants were excluded. One of them had the totally opposite judgment on facing direction attribute, and the other one could not discriminate emotional state in more than half of the trials.

#### fMRI experiment

In the fMRI scanner, stimuli were back-projected onto a screen (60 Hz frame rate, 1,024 × 768 pixels screen resolution) via a liquid crystal projector and viewed through a mirror mounted on the head coil. fMRI runs were arranged in a block design with each run containing 24 blocks. Each block consisted of a BM sequence lasting 12 s. In every 2 s, the BM stimulus had a spatial jitter of 1^o^ to reduce the potential adaptation effect (Dubois et al. 2015). Eight BM sequences were repeated three times in random order and were interleaved by 6 s fixation blocks. Participants completed six runs, and each run lasted 7 min and 18 s. To ensure that the participant’s attention was focused on the stimulus during scanning, the BM sequence was either forward-walking or backward-walking and randomly changed 1—2 times in each block (Fig. 1B). The participants were required to detect how many times the walking direction of the BM sequence had changed and answered by pressing one of the two keys on a keyboard after the stimulus disappeared. All participants demonstrated good performance, with a mean accuracy of 0.883 ± 0.013 and an average reaction time of 1.004 ± 0.054 s. An additional group of participants evaluated the facing direction, gender, and emotional state attributes when the BM sequence was forward-walking or backward-walking, which verified that walking directions did not significantly influence the recognition of these attributes (see Supplementary Material Table S2 for details).

### fMRI acquisition

Functional and anatomical data were collected using a 3-Tesla Siemens Prisma scanner at the Beijing Magnetic Resonance Imaging Center for Brain Research. Functional data were collected using a T2^*^-weighted echo planar imaging sequence with the following parameters: 78 axial slices (with multiband), repetition time (TR) = 2,000 ms, echo time (TE) = 30 ms, flip angle = 70^o^, field of view (FOV) = 192 × 192 mm^2^, matrix size = 96 × 96, thickness/gap = 2/0 mm. Each fMRI session consists of 219 functional volumes. A 3D T1-weighted magnetization-prepared rapid gradient echo (MP-RAGE) image was acquired with the following parameters: 128 sagittal slices, TR = 2,600 ms, TE = 3.02 ms, inversion time (TI) = 900 ms, flip angle = 8^o^, FOV = 256 × 224 mm^2^, matrix size = 256 × 224, slice thickness/gap = 1/0 mm.

### Preprocessing and general linear model analysis

The fMRI data were preprocessed using statistical parametric mapping 12 (SPM12, http://www.fil.ion.ucl.ac.uk/spm/, Wellcome Center for Human Neuroimaging, London, United Kingdom). The first three volumes were discarded to avoid T1 saturation. Then the functional images were corrected for slice acquisition time and head motion, and co-registered with the T1 anatomical image. Next, anatomical images were spatially normalized to the Montreal Neurological Institute template, and normalization parameters were applied to the functional images.

After preprocessing, we applied traditional general linear model (GLM)-based analysis. For each participant and each run, beta weights of the experimental conditions were estimated using design matrices containing predictors of the eight stimuli and six head motion parameters in a gray matter mask. An absolute threshold masking value of 0.2 was applied to avoid possible edge effects between different tissue types (Ashburner 2007). The resulting SPM_T_ images for the eight stimuli were used for RSA (Salmela et al. 2018). The beta weights for 8 stimuli × 6 runs were used for the MVPA (Turner et al. 2012).

### Multiple regression RSA

To investigate which brain areas were in response to the three attributes of BM, we employed a multiple regression RSA with a searchlight approach using CoSMoMVPA toolbox (Oosterhof et al. 2016). Firstly, we created theoretical RDMs for each attribute of the stimuli (Fig. 1C). Each RDM was an 8 × 8 binary matrix in which 1 corresponded to a between-category stimulus comparison (e.g. male vs. female for gender discrimination) and 0 corresponded to a within-category stimulus comparison (e.g. female vs. female for gender discrimination). This procedure resulted in three theoretical RDMs corresponding to facing direction, gender, and emotional state attributes of the stimuli. To exclude the influence of low-level visual properties such as retinotopic shape biases across our stimulus categories, we added another predictor based on a model of V1 cortical neurons to the multiple regression RSA. We used the C1 units of hierarchical max-pooling (HMAX) model (Riesenhuber and Poggio 1999) to simulate every BM sequence as the V1 cortical response using software provided by the Center for Biological & Computational Learning at Massachusetts Institute of Technology (http://cbcl.mit.edu/software-datasets/). Because processing by the units is approximated as essentially instantaneous in HMAX model (Serre et al. 2007), we first modeled each frame of the BM sequence and then averaged the response of each frame. Then we derived the V1 RDM using 1—Pearson correlation as a measure of dissimilarity (Fig. 1D). Additionally, we analyzed the correlations between three theoretical RDMs and V1 RDM (Fig. 1). The correlation between V1 RDM and the theoretical RDMs is 0.21 for facing direction, 0.68 for gender, and 0.27 for emotion, respectively.

Next, we calculated the neural RDM. In each searchlight, we created a neural representational similarity matrix (RSM) by calculating the Pearson correlation between the SPM_T_ maps from every item pair and then converting the neural RSM into neural RDM using 1—Pearson correlation. After that, we performed the multiple regression RSA using the three theoretical RDMs and the V1 RDM as the independent variables and the neural RDM as the dependent variable. This method can identify the brain maps for the processing of one attribute and, meanwhile, exclude the influence from the other attributes and low-level visual properties. We conducted the multiple regression RSA for each participant within the gray matter mask using a searchlight approach, with each searchlight consisting of 200 voxels (Anderson et al. 2015; Thornton and Mitchell 2017; Liuzzi et al. 2020). The resulting correlation values for each predictor were assigned to the central node of each searchlight, leading to a correlation map for each attribute, separately for each participant. Additionally, we conducted an RSA only with the V1 RDM as a predictor to verify that this model represents the primary visual cortex activity for the BM sequence (see Supplementary Material Fig. S1 for details). We also conducted an RSA with the three theory RDMs but without the V1 RDM as predictors. Results showed similar cortical maps for each attribute except the visual network (Fig. S2 in Supplementary Material). All RSA results were Fisher transformed and then conducted one-sample *t*-tests. Statistical maps were corrected for multiple comparisons using a cluster-based Monte Carlo simulation algorithm as implemented in the CoSMoMVPA toolbox. We used a threshold of *P* = 0.001 at the initial voxel-wise, and 5,000 iterations of Monte Carlo simulations (Forman et al. 1995; Goebel et al. 2006). For visualization, maps were projected on a cortex surface of the BrainNet toolbox (Xia et al. 2013).

### Scrambled RDM-based RSA and dice coefficient analysis

We further investigated how the neural encoding of an attribute is influenced by that of the other two attributes. To do this, we carried out a new set of multiple regression RSAs for the three attributes, respectively. In these RSAs, the target theoretical RDM remained unchanged, and the other two theoretical RDMs were shuffled randomly (Carlson et al. 2013; Liang et al. 2013; Bayet et al. 2020). For example, for the gender attribute, we scrambled the facing direction and emotional state RDMs but retained the theoretical RDM of gender, then recalculated the multiple regression RSA with the searchlight approach. This process was repeated 1,000 times for each attribute and each participant, and the resulting correlation map was transformed into a z-score. Then all the z maps were averaged for each attribute and each participant. After that, the results were entered into one-sample *t*-tests for each attribute, respectively, and corrected by 5,000 iterations of Monte Carlo simulations, resulting in the group-level maps. Note that the above brain map each reflected the processing of an attribute without excluding the implied influence from the processing of the other attributes.

Then we used the Dice coefficient (DC; Dice 1945) to assess the degree of overlap between the group-level statistic maps of the multiple regression RSA and the RSA with scrambled RDM for each attribute (Gorgolewski et al. 2013; Sair et al. 2016). The formula of DC is 2 × N_C_/(N_1_ + N_2_), where N_C_ is the number of voxels the two statistics maps share in common, N_1_ is the number of voxels in the first map, and N_2_ is the number of voxels in the second map. DC ranges from 0 to 1, where 1 indicates complete congruence between the number and location of voxels in both threshold maps, while 0 indicates no congruence (Dice 1945; T. A. Sørensen 1948). We used this analysis to evaluate the extent to which the neural encoding of one attribute is modulated by that of the other two. Specifically, if the processing of an attribute is completely not influenced by the others, the brain maps of the multiple regression RSA and the RSA with scrambled RDM would perfectly overlap, leading to a DC of 1. On the contrary, if the processing of an attribute is dependent with the processing of the others, the brain maps of the multiple regression RSA and the RSA with scrambled RDM would have no overlapped areas, leading to a DC of 0. Between 0 and 1, the smaller the influence of other attributes, the larger the DC value will be.

### Multivoxel pattern analysis

We used MVPA to measure whether the significant clusters identified by multiple regression RSA were specific to the neural encoding of the corresponding BM attributes. We first extracted the beta map of 8 stimuli × 6 runs from GLM analysis, resulting in a total of 48 beta maps for each participant. These beta maps were split into two parts corresponding to the categories for facing direction (left vs. right), gender (male vs. female), and emotional state (happy vs. sad), respectively. For example, when classifying gender, the 48 beta maps were split into 24 female beta maps and 24 male beta maps. Then we conducted the MVPA for each attribute and each participant within the gray matter mask using a searchlight approach, with each searchlight consisting of 200 voxels. On each searchlight, we demeaned the data by subtracting the mean beta value from each beta value of the individual voxel to reduce the amplitude effects of different conditions. We used a leave-one-run-out cross-validation method, so that for each iteration, we trained a linear support vector machine (Chang and Lin 2011) classifier using data from five fMRI runs and tested the classifier with the data from the one remaining run. After that, a whole-brain map for each participant was defined in which the center voxel of each searchlight was labeled according to classification accuracy, and then the classification accuracy of each cluster identified by multiple regression RSA was defined as the mean classification accuracy of all voxels located in this cluster (the whole-brain searchlight MVPA results were presented in Supplementary Material Fig. S3). The clusters’ accuracies were entered into a one-sample *t*-test against chance (50%, Fuelscher et al. 2019; Lee et al. 2012; Sapountzis et al. 2010). We also used paired *t*-test to compare the clusters’ accuracies between the two attributes pairs and corrected the results by FDR correction (*P* < 0.05). Results were projected on a cortex surface of the BrainNet toolbox on three levels: (i) the classification accuracy of the corresponding attribute is significantly above chance level, (ii) the classification accuracy of one attribute is significantly higher than one of the other attributes, (iii) the classification accuracy of one attribute is significantly higher than both of the other two attributes.

### RSA between neural and behavioral RDMs

We investigated whether the fMRI results were related to behavioral judgments. To this end, we used the responses obtained in the behavioral experiment to define the behavioral RDM for each participant. We derived the behavioral RDM of each attribute by using the Euclidean distance as the distance metric of the judgments between each pair of stimuli. Then we conducted an RSA with behavioral RDMs as predictors in each cluster identified in multiple regression RSA for each participant, respectively. Then, the results were Fisher transformed and entered into a one-sample *t*-test and corrected by FDR correction (*P* < 0.05) across clusters.

## Results

### Brain networks for encoding different BM attributes

**Fig. 2:**
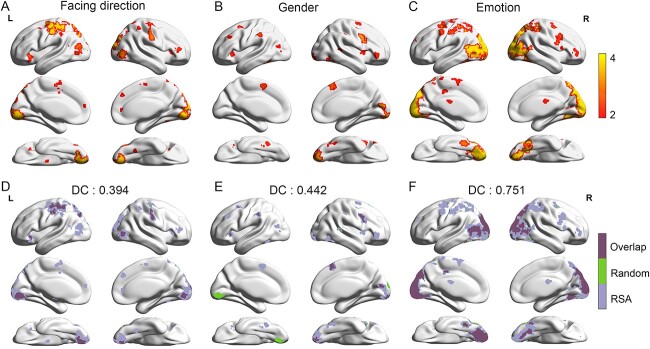
RSA results of multiple regression and random results. Group-level results of the searchlight-based multiple regression RSA of facing direction (A), gender (B), and emotional state (C). The group-level results of 1,000 times random RSA results of facing direction (D), gender (E), and emotional state (F). The light purple color shows the results of multiple regression RSA. The green color shows the random RSA results. The dark purple color shows the overlapping results of multiple regression RSA and random RSA. DC between the multiple regression RSA maps and the random RSA maps. Statistical maps show the clusters after Monte Carlo simulations (*P* = 0.001 at the initial voxel-wise, 5,000 iterations).

The multiple regression RSA revealed the brain regions encoding facing direction, gender, and emotional state (Fig. 2; corrected by a cluster-based Monte Carlo simulation with a threshold of *P* = 0.001 at the initial voxel-wise and 5,000 iterations). Specifically, the brain regions encoding the facing direction information involved the bilateral lingual gyri (Brodmann areas [BA] 17), left middle occipital gyrus (MOG, BA 37), right supper occipital gyrus (SOG, BA18), bilateral FG (BA 18), bilateral middle temporal gyri (MTG, BA 19), left inferior parietal lobules (IPL, BA 40), bilateral superior parietal lobes (SPL, BA 7), bilateral postcentral gyri (BA 3), bilateral precentral gyri (BA 4), left supplementary motor area (SMA, BA 6), bilateral insula (BA 13), bilateral IFG (BA 47), and right anterior cingulate cortex (ACC, BA 10; Fig. 2A). The brain regions encoding the gender information involved the right lingual gyrus (BA 17), bilateral FG (BA 37), right inferior temporal gyrus (ITG, BA 37), left MTG (BA 19), right pSTS (BA 22), left IPL (BA 40), bilateral postcentral gyri (BA 2), right SMA (BA 6), bilateral insula (BA 13), right IFG (BA 47), and bilateral MFG (BA 6, Fig. 2B). The brain regions encoding the emotional state information involved the bilateral lingual gyri (BA 17, 18), bilateral IOG (BA 18), bilateral MOG (BA 19), bilateral SOG (BA 7, 18, 19), bilateral FG (BA 19), bilateral ITG (BA 37), bilateral MTG (BA 39), bilateral IPL (BA 40), bilateral SPL (BA 7), bilateral postcentral gyri (BA 3), bilateral precentral gyri (BA 6), left SMA (BA 6), right IFG (BA 46), bilateral MFG (BA 6), and right SFG (BA 11), left putamen (BA 48, Fig. 2C). These results together demonstrated that the respective neural encoding of the facing direction, gender, and emotional state information embedded in BM stimuli involved considerably overlapping brain regions, which raised the possibility that the neural representations of the three attributes of BM might be shared and possibly interact with each other.

### Hierarchical neural encoding among the three BM attributes

We further investigated how the neural encoding of an attribute is influenced by that of the other attributes. To do this, for each attribute, we conducted a new searchlight multiple regression RSA in which the theoretical RDM of the target attribute remained unchanged, and the other two theoretical RDMs were scrambled. This procedure was repeated 1,000 times and then the results were averaged. If the neural encoding of the target attribute is influenced by the processing of the other two attributes, then the beta value for the target attribute would be different between the RSA results before and after scrambling manipulation, resulting in a variation in the identified statistic maps by the searchlight approach. DCs evaluated the extent to which the neural encoding of one attribute is modulated by that of the other two. Figure 2 shows the DC for the pairs of analyses before and after scrambling manipulation on group-level threshold statistic maps. The DC is 0.394 for facing direction (Fig. 2D), 0.442 for gender (Fig. 2E), and 0.751 for emotional state (Fig. 2F). These results demonstrated that the neural encodings of all attributes were influenced by each other to some extent, that is, the neural encodings of the three attributes were recurrently interactive. Among the three attributes, the variations of their neural encodings, inversely related to the DC values, before and after scrambling manipulation were in descending order for facing direction, gender, and emotional state.

### Brain areas specific to the classifications of BM attributes

**Fig. 3:**
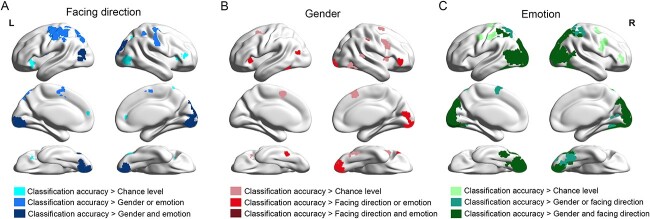
MVPA results in the significant clusters defined in RSA of facing direction (A), gender (B), and emotional state (C). The light colors mean the classification accuracy was significantly higher than chance level (50%). The medium colors mean the classification accuracy was significantly higher than one of the other attributes. The dark colors mean the classification accuracy was significantly higher than both of the other two attributes. Results were corrected by FDR correction (*P* < 0.05).

To further explore whether the brain areas in the BM attribute encoding networks were specific to the corresponding attribute classifications, we performed MVPA on the clusters revealed in RSA to measure the classification accuracies for discriminating the three attributes. On most clusters, the classification accuracies for the corresponding attribute were significantly above chance level (50%). A portion of the brain areas showed significantly higher classification accuracies for the corresponding attribute than for the other attributes. We displayed the statistical results on brain maps (Fig. 3). In the facing direction encoding network, classification accuracies were significantly above chance level in the bilateral lingual gyri (BA 17), left MOG (BA 37), right SOG (BA 18), bilateral MTG (BA 19), bilateral FG (BA 18), bilateral SPL (BA 7), bilateral postcentral gyri (BA 3), left precentral gyrus (BA 4), left SMA (BA 6), bilateral insula (BA 13), right IFG (BA 47), and right ACC (BA 10, *P* < 0.05, FDR correction). Among these regions, the classification accuracies of facing direction were significantly higher than one of the other two attributes in the left MOG (BA 37), left SPL (BA 37), bilateral postcentral gyri (BA 3), left precentral gyrus (BA 4), left SMA (BA 6), and left insula (BA 13), and significantly higher than both of the other two attributes in the left MTG (BA 19), right SOG (BA 18), bilateral lingual gyri (BA 17), and bilateral FG (BA 18). In the gender encoding network, classification accuracies were significantly above chance level in the right lingual gyrus (BA 17), left FG (BA 37), right ITG (BA 37), left MTG (BA 19), right pSTS (BA 22), right postcentral gyrus (BA 2), right SMA (BA 6), bilateral insula (BA 13), right IFG (BA 47), and right MFG (BA 6, *P* < 0.05, FDR correction). Among these regions, the classification accuracies of gender were significantly higher than one of the other two attributes in the right lingual (BA 17), left FG (BA 37), left MTG (BA 19), right postcentral gyrus (BA 2), left insula (BA 13), and right IFG (BA 47), but no cluster has significantly higher classification accuracies than both of the other two attributes. In the emotional state encoding network, classification accuracies were significantly above chance level in the bilateral lingual gyri (BA 17, 18), bilateral IOG (BA 18), bilateral MOG (BA 19), bilateral SOG (BA 7, 18, 19), bilateral FG (BA 19), bilateral ITG (BA 37), bilateral MTG (BA 39), bilateral IPL (BA 40), bilateral SPL (BA 7), bilateral postcentral gyri (BA 3), bilateral precentral gyri (BA 6), left SMA (BA 6), right SFG (BA 11), right MFG (BA 6), and right IFG (BA 46, *P* < 0.05, FDR correction). Among these regions, the classification accuracies of emotional state were significantly higher than one of the other two attributes in the left postcentral gyrus (BA 3), left SMA (BA 6), and right FG (BA 19), and significantly higher than both of the other two attributes in the bilateral lingual gyri (BA 17, 18), bilateral IOG (BA 18), bilateral MOG (BA 19), bilateral SOG (BA 7, 18, 19), left FG (BA 19), left ITG (BA 37) bilateral MTG (BA 39), and bilateral SPL (BA 7).

### Correlation between neural representations and behavioral results

**Fig. 4:**
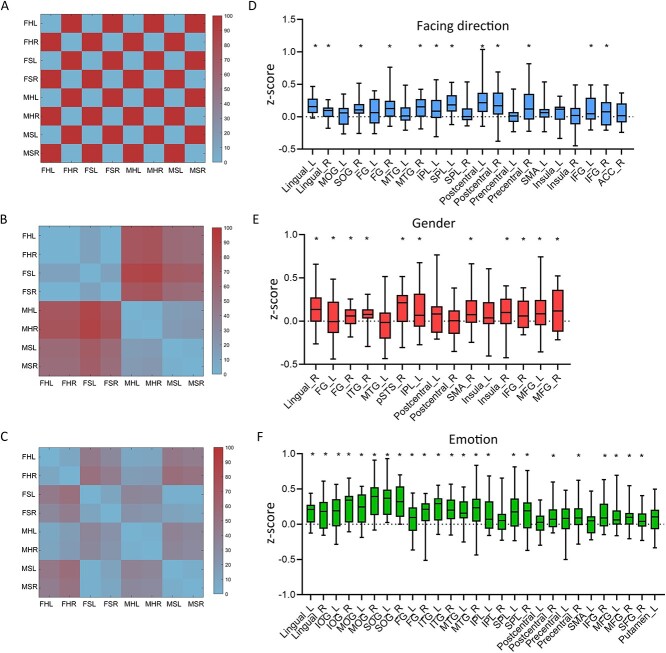
Correlations between behavioral and neural RDMs on the significant clusters defined in RSA. Behavioral RDM for facing direction (A), gender (B), and emotional state (C) of a representative participant. The correlation results for facing direction (D), gender (E), and emotional state (F). Results were Fisher transformed. ^*^P < 0.05 after FDR correction.

Not all information that can be read out from brain activity is directly used by the brain to guide behaviors. To investigate whether the brain regions revealed by RSA were correlated with participants’ behavioral discrimination of each attribute, we calculated the correlation between behavioral RDMs and neural RDMs in the clusters identified in the multiple regression RSA. Figure 4 shows the correlation on each cluster (*P* < 0.05, FDR correction). Most of the clusters showed significant correlations between behavioral and neural responses.

## Discussion

The present study investigated the brain encoding of multidimensional BM information. Using multiple regression RSA, we identified distributed brain networks related to facing direction, gender, and emotional state processing. These brain areas were governed by a recurrently interactive mechanism that the processing of each attribute was influenced by the others, leading to a hierarchical structure: the respective neural encoding of facing direction, gender, and emotional state is modulated by each other in descending order. Among the three attribute encoding networks, a portion of the brain areas was specific to the classification of the corresponding attribute. Most of these areas showed significant correlations between behavioral and neural responses. Taken together, these results revealed the distributed and hierarchical brain mechanisms of multidimensional BM processing and provided constraints on computational models of BM perception.

### Recurrently interactive multidimensional BM attribute encoding

While a few prior studies have investigated the brain substrates underlying particular BM attribute perception or its cognitive effect (De Lussanet et al. 2008; Michels et al. 2009; Atkinson et al. 2012; Alaerts et al. 2014; Goldberg et al. 2015; Jastorff et al. 2015; Poyo Solanas et al. 2020), they have focused on the processing of a single attribute of BM, and hence could not address in what order different BM attributes are encoded in the brain and how they interact with each other. The current study extended previous findings and, for the first time, investigated the brain network related to the neural encoding of BM attributes from a multidimensional perspective. Although different BM attributes convey different levels of information, the current study demonstrated that the neural encodings of different BM attributes are recurrently interactive. It has been long assumed that low-level and simple features are extracted early in the visual system while high-level and complex attributes are processed at a relatively late stage of visual processing (Rousselet et al. 2004). Among BM attributes, facing direction conveys relatively “low-level” orientation information, which can be similarly transmitted by a lot of inanimate objects such as moving cars or moving balls. Gender conveys biological or physiological information that is specific to living beings. The emotional state represents relatively “high-level” mental state information. Recently, researchers have proposed a dual-process theory for BM perception, which suggests that detecting facing direction is a rapid preattentive process while evaluating gender and emotional state requires more cognitive processes (Hirai and Senju 2020). However, our DC analysis demonstrates that the processes of BM attributes are recurrently influenced by each other rather than in a simply independent or feedforward manner.

The DC analysis also reveals a hierarchical structure among the three attributes. The DC for facing direction is minimum, suggesting its neural encoding is more influenced by the processing of the other attributes. The DC for emotional state is maximum, suggesting its neural encoding is less influenced by the processing of the other attributes. This pattern of results is in accordance with previous behavioral findings on BM perception. Although it has long been thought that the gender information of both the agent and the observer could affect the perception of emotional state (Cutting and Kozlowski 1977; Johnson et al. 2011; Kret et al. 2011), researchers also found that the emotional state of BM could affect the gender judgment (Johnson et al. 2011). Furthermore, the gender of the BM stimulus has been shown to modulate the perception of its facing direction (Yang et al. 2014).

### Brain network as a function of BM attribute extraction

This study revealed extensive networks involved in extracting facing direction, gender, and emotional state from BM. Among these networks identified by RSA, MVPA further confirmed that the classification accuracies for the corresponding attributes were significantly above chance level in a bulk of areas. More importantly, in some areas, the classification accuracies for the corresponding attribute were significantly higher than the other attributes, implying that the neural responses were more distinguishable for the corresponding attribute. On the other hand, to further explore whether the fMRI signals related to behavioral results, we correlated neural RDMs to behavioral RDMs and found that most of the brain areas in the networks identified by RSA showed significant correlations. This pattern of results provides a preliminary link between the behavioral performance and the corresponding neural substrates for processing BM attributes.

Among the facing direction encoding network, the roles of the lingual gyri, SOG, FG, SPL, postcentral gyri, and IFG seem to be crucial. MVPA revealed that the facing direction classification accuracies were significantly above chance level in the right IFG, higher than one of the other attributes in the left SPL and bilateral postcentral gyri, and higher than the other two attributes in the bilateral lingual gyri, right SOG, and bilateral FG. The neural responses of all the above regions were significant correlated with behavioral results. Additionally, in our results, the bilateral FG, bilateral postcentral gyri, and bilateral IFG have been reported in previous studies on BM facing direction (De Lussanet et al. 2008; Michels et al. 2009). These results share a partially overlapping brain network with those obtained from face and body perception. The brain regions representing the facing direction of bodies involved the FG (Taylor et al. 2010; Vangeneugden et al. 2012; Bellot et al. 2021) and pSTS (Vangeneugden et al. 2014). Recently, researchers have demonstrated a stimulus-independent neural code for the facing direction of face and body in the occipitotemporal cortex, including the occipital face area, extrastriate body area, lateral occipital complex, and early visual cortex (Foster et al. 2022). These brain regions are close to the FG and MTG in the facing direction network observed in the current study. Besides, considering the finding in the previous electroencephalography (EEG) study that the facing direction of BM can trigger an early directing attention negativity in the occipito-parietal electrodes (PO5/6 and PO7/8; Wang et al. 2014), we highlight the role of SPL. Moreover, previous case studies have also provided evidence that patients with parietal lobe lesions have difficulty in discriminating the facing direction of BM, but not the direction of low-level motion (Battelli et al. 2003).

Among the gender encoding network, MVPA revealed that in the right pSTS, left FG, left MTG, left insula, and right lingual gyrus, the gender classification accuracies were significantly above chance level and higher than one of the other attributes. The right lingual gyrus, left FG, and left MFG also encoded the similarity patterns revealed by the behavioral experiment. These results share a partially overlapping brain network with previous results on face and body processing. It is reported that the brain regions representing the gender of face and body include the FG (Contreras et al. 2013), pSTS, insula, and SMA (Kret et al. 2011).

Among the emotional state encoding network, the MOG, ITG, MTG, and SPL might be crucial. MVPA revealed that in the bilateral MOG, ITG, MTG, and SPL, the emotional state classification accuracies were significantly above chance level and higher than other attributes. In the bilateral MOG, bilateral ITG, bilateral MTG, and bilateral SPL, the correlations between neural responses and behavioral results were significant. Additionally, the FG, ITG, MTG, precentral gyrus, and IFG have also been reported in previous studies (Jastorff et al. 2015; Ross et al. 2019; Poyo Solanas et al. 2020). Precious studies have revealed a broad network for emotional state processing on other socially relevant information. Concentrated on the studies about happy and sad emotions, we concluded that the brain regions processing emotional information include the MFG (Kesler-West et al. 2001; McLellan et al. 2012), dorsolateral prefrontal cortex (Vanderhasselt et al. 2011), SFG, IFG (McLellan et al. 2012), ACC (Yoshino et al. 2010), amygdala (Gaffrey et al. 2011), and insula (Hall et al. 2014). It seems that emotional state processing involves complex neural mechanisms that are influenced by many factors, such as emotion types and the expressing moods.

### A distributed and hierarchical neural network for BM processing

To the best of our knowledge, three main neural models have been proposed to account for BM processing. Giese and Poggio (2003) proposed a hierarchical and feedforward computational model with two parallel pathways for the processing of the form and motion of BM. Both pathways consist of several levels and finally converge at the STS. This model does not involve the processing of different BM attributes. Lange and Lappe (2006) proposed a two-stage computational model. The first stage analyzes the form information in each frame, then the second stage analyzes the temporal order of the selected frames, leading to the output on the global motion aspects of the stimulus. This model can well explain the processing of facing direction, but hardly expand to the processing of other attributes. Hirai and Senju (2020) proposed a two-process model for BM perception. While this model supposed that the first system detects foot motion (thus process facing direction) and the second system evaluates global bodily actions (thus process gender and emotional state), the main purpose of the model was to separate the rapid, subcortical system from the slow, cortical system. Here we developed a theoretical model for multidimensional BM processing based on our results and the findings from previous studies.

**Fig. 5:**
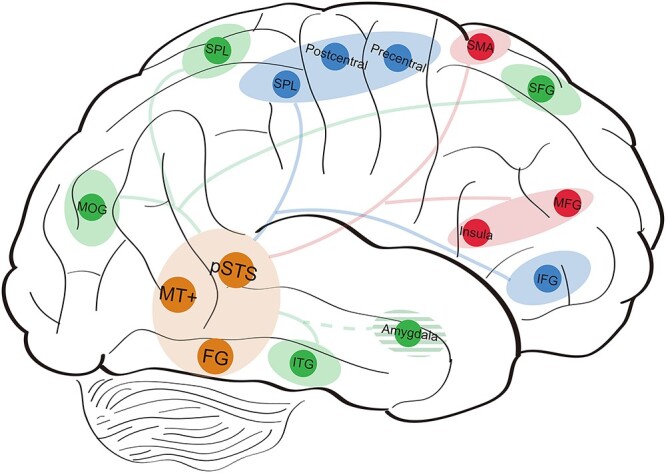
The model of a distributed and hierarchical system for BM attribute representations. The core system (in orange color) is composed of pSTS, MT+, and FG. The extended system for facing direction representation (in blue color) is composed of SPL, postcentral gyrus, precentral gyrus, and IFG. The extended system for gender representation (in red color) is composed of SMA, insula, and MFG. The extended system for emotional state representation (in green color) is composed of MOG, ITG, SPL, amygdala, and SFG.

BM perception, as a crucial visual process for socially relevant information, is analogous to face perception, which is accomplished by a dynamic and hierarchical neural system (Giese and Poggio 2003; Hirai and Senju 2020). Following the model of distributed neural system for multidimensional face perception (Haxby et al. 2000), we propose a model that mediates the perception of multidimensional BM information (Fig. 5). The model has a branching structure: a core system for the visual analysis of BM is distinguished from an extended system that processes the attributes gleaned from BM. The core system comprises three brain regions: the pSTS, the MT+, and the FG. The current study demonstrates that these regions are substantially involved in the neural representations of all the three BM attributes. Anatomical configuration suggests a hierarchical organization among these regions, in which the middle temporal cortex may provide input to the lateral FG and STS (Gauthier and Logothetis 2000; Cross et al. 2010). Functional connectivity studies of BM processing suggest that the right FG, MT+, and STS are functionally integrated (Dasgupta et al. 2017; Sokolov et al. 2018). Among the core system, the pSTS is the core brain area dedicated to BM processing (Allison et al. 2000; Blake and Shiffrar 2007; Yovel and O’Toole 2016), whose causal role has been assessed in brain stimulation (Grossman et al. 2005; van Kemenade et al. 2012) and brain lesion (Saygin 2007) studies. The pSTS not only selectively responds to BM compared with non-BM stimuli (Bonda et al. 1996; Howard et al. 1996; Chang et al. 2018), but also exhibits special responses across different types of BM stimuli (Frith and Frith 2010). Accumulating evidence also suggests the critical functions of MT+ (Herrington et al. 2007; Jastorff and Orban 2009) and FG (Vaina et al. 2001; Peelen et al. 2006; Lichtensteiger et al. 2008; Michels et al. 2009; Sokolov et al. 2018) in BM processing. The MT+ is a vital region for analyzing the kinematic cues of BM (Jastorff and Orban 2009), while the FG plays a key role in body form processing (Thompson and Parasuraman 2012).

The extended system consists of distinct brain regions for further processing the different BM attributes, in concert with other neural networks. The neural network for processing facing direction includes the SPL, postcentral and precentral gyri, and IFG. The neural activity in the precentral and postcentral gyri showed higher classification accuracies for facing direction than the other attributes and correlated with behavioral results. Moreover, the facing direction of BM is an important social cue that can induce a reflexive attentional orienting effect in the occipito-parietal region (Shi et al. 2010; Wang et al. 2014). Facing direction thus provides a basis for implying the goal of an individual, which might be extracted in the IFG (De Lussanet et al. 2008; Thompson and Parasuraman 2012). The neural network for processing gender includes the SMA, insula, and MFG. These brain regions were revealed by the present study as well as reported in previous studies on gender perception (de Gelder et al. 2010; Kret et al. 2011). The neural network for processing emotional state includes the MOG, ITG, SPL, SFG, and amygdala. The ITG is identified as a relevant region for the perception of human bodies and is directly neighbored by the FG (Weiner and Grill-Spector 2011), and shows increased activity in response to emotional body expressions (de Gelder et al. 2004; Prochnow et al. 2013). The SPL is found to be activated by the visual stimulation of socio-emotional stimuli (de Gelder et al. 2015). SFG is reported in previous studies on emotional state perception (Mak et al. 2009; McLellan et al. 2012). In the current study, the SFG was identified in multiple regression RSA for emotional state and correlated with behavioral results. The amygdala is considered a hub region for emotional processing (Phelps 2006; Sergerie et al. 2008; Andrewes and Jenkins 2019). Therefore, we take it into the emotional state representation network in the extended system. However, our RSA did not reveal the amygdala in emotional BM processing, which is likely due to that the emotions employed in the current study were happy and sad, and did not evoke strong neural activation in the amygdala. Previous studies that reported amygdala activations in BM emotional processing usually adopted fearful or angry emotions (Jastorff et al. 2015; Poyo Solanas et al. 2020). Therefore, further work is required to employ a variety of emotion types to verify the emotional state representation network for BM perception.

Our model is the first to consider the neural mechanism for the processing of BM attributes. Previous models primarily described neural processing from the perception of basic visual features (e.g. form, local motion) to the recognition of integrated BM information. The advantage of our model is that it provides a different perspective for BM processing, which concentrates on the neural encodings of distinct physical, biological, and social attributes from BM signals. Future directions may include analyzing the functional connections of distinct BM attribute representations in the model and linking them with behavioral performance, as well as further testing the model's predictive capabilities.

### Study limitations

The main limitation of the present study was that only three BM attributes were employed. The human visual system can extract and process many other attributes of BM, such as identity (Ng et al. 2006; Westhoff and Troje 2007; Baragchizadeh and O’Toole 2017), personality trait (Klüver et al. 2016), age (Montepare and Zebrowitz-McArthur 1988), and familiarity (Hahn and O’Toole 2017). Thus, further studies could simultaneously consider more attributes to investigate how the brain encodes distinct physical, biological, and social attributes from BM. Besides, a larger sample size may be necessary for a study employing more BM attributes.

While we discovered a hierarchical structure for processing multidimensional BM, we were unable to determine how the processing of distinct BM attributes unfolds over time due to the limited temporal resolution of fMRI. Further studies utilizing techniques such as magnetoencephalography (MEG) or EEG could shed light on the temporal characteristics of these processes.

Our results revealed significant correlations between behavioral and neural responses in most of the regions of the three BM attributes networks. However, the current behavioral experiment had two limitations. Firstly, the behavioral results relied on subjective evaluation of BM attributes, which might be influenced by individuals' judgment criteria. Future studies could employ an objective BM attribute discrimination paradigm. Secondly, the behavioral experiment was conducted before the fMRI scanning, and a more direct link between behavioral and neural responses could be obtained from concurrent behavioral responses during fMRI scanning.

## Conclusions

The brain representations of multidimensional BM engage a distributed and hierarchical network, which consists of a core system (i.e. the pSTS, MT+, and FG) and an extended system that processes the distinct attributes of BM. The neural encodings of different BM attributes are governed by a recurrently interactive mechanism that the processing of each attribute is influenced by the others, leading to a hierarchical structure in which the respective neural representation of facing direction, gender, and emotional state is modulated by each other in descending order.

## Data availability

Anonymized data, codes, and the stimuli associated with this work are available at http://ir.psych.ac.cn/handle/311026/43299.

## Supplementary Material

SupplementaryMaterial_bhad136

## References

[ref1] Alaerts K, Woolley DG, Steyaert J, Di Martino A, Swinnen SP, Wenderoth N. Underconnectivity of the superior temporal sulcus predicts emotion recognition deficits in autism. Soc Cogn Affect Neurosci. 2014:9:1589–1600. 24078018 10.1093/scan/nst156 PMC4187281

[ref2] Allison T, Puce A, McCarthy G. Social perception from visual cues: role of the STS region. Trends Cogn Sci. 2000:4:267–278. 10859571 10.1016/s1364-6613(00)01501-1

[ref3] Anderson AJ, Bruni E, Lopopolo A, Poesio M, Baroni M. Reading visually embodied meaning from the brain: visually grounded computational models decode visual-object mental imagery induced by written text. NeuroImage. 2015:120:309–322. 26188260 10.1016/j.neuroimage.2015.06.093

[ref4] Andrewes DG, Jenkins LM. The role of the amygdala and the ventromedial prefrontal cortex in emotional regulation: implications for post-traumatic stress disorder. Neuropsychol Rev. 2019:29:220–243. 30877420 10.1007/s11065-019-09398-4

[ref5] Ashburner J . A fast diffeomorphic image registration algorithm. NeuroImage. 2007:38:95–113. 17761438 10.1016/j.neuroimage.2007.07.007

[ref6] Atkinson AP, Dittrich WH, Gemmell AJ, Young AW. Emotion perception from dynamic and static body expressions in point-light and full-light displays. Perception. 2004:33:717–746. 15330366 10.1068/p5096

[ref7] Atkinson AP, Vuong QC, Smithson HE. Modulation of the face- and body-selective visual regions by the motion and emotion of point-light face and body stimuli. NeuroImage. 2012:59:1700–1712. 21924368 10.1016/j.neuroimage.2011.08.073

[ref8] Baragchizadeh A, O’Toole A. Identity matching of unfamiliar people from point-light biological motion. J Vision. 2017:17:62.

[ref9] Battelli L, Cavanagh P, Thornton IM. Perception of biological motion in parietal patients. Neuropsychol Neuropsychol Motion Perception. 2003:41:1808–1816. 10.1016/s0028-3932(03)00182-9 14527544

[ref10] Bayet L, Zinszer BD, Reilly E, Cataldo JK, Pruitt Z, Cichy RM, Nelson CA, Aslin RN. Temporal dynamics of visual representations in the infant brain. Dev Cogn Neuros-neth. 2020:45:100860. 10.1016/j.dcn.2020.100860 PMC7498752 32932205

[ref11] Bellot E, Abassi E, Papeo L. Moving toward versus away from another: how body motion direction changes the representation of bodies and actions in the visual cortex. Cereb Cortex. 2021:31:2670–2685. 33401307 10.1093/cercor/bhaa382

[ref12] Blake R, Shiffrar M. Perception of human motion. Annu Rev Psychol. 2007:58:47–73. 16903802 10.1146/annurev.psych.57.102904.190152

[ref13] Bonda E, Petrides M, Ostry D, Evans A. Specific involvement of human parietal systems and the amygdala in the perception of biological motion. J Neurosci. 1996:16:3737–3744. 8642416 10.1523/JNEUROSCI.16-11-03737.1996 PMC6578830

[ref14] Brainard DH . The psychophysics toolbox. Spatial Vis. 1997:10:433–436. 9176952

[ref15] Carlson T, Tovar DA, Alink A, Kriegeskorte N. Representational dynamics of object vision: the first 1000 ms. J Vis. 2013:13:1–1. 10.1167/13.10.1 23908380

[ref16] Chang C-C, Lin C-J. LIBSVM: a library for support vector machines. ACM Trans Intell Syst Technol. 2011:2:1–27.

[ref17] Chang DHF, Ban H, Ikegaya Y, Fujita I, Troje NF. Cortical and subcortical responses to biological motion. NeuroImage. 2018:174:87–96. 29524623 10.1016/j.neuroimage.2018.03.013

[ref18] Clarke TJ, Bradshaw MF, Field DT, Hampson SE, Rose D. The perception of emotion from body movement in point-light displays of interpersonal dialogue. Perception. 2005:34:1171–1180. 16309112 10.1068/p5203

[ref19] Contreras JM, Banaji MR, Mitchell JP. Multivoxel patterns in fusiform face area differentiate faces by sex and race. PLoS One. 2013:8:e69684. 23936077 10.1371/journal.pone.0069684 PMC3729837

[ref20] Cross ES, Mackie EC, Wolford G, de C. Hamilton AF. Contorted and ordinary body postures in the human brain. Exp Brain Res. 2010:204:397–407. 19943038 10.1007/s00221-009-2093-x PMC2895886

[ref21] Cutting JE, Kozlowski LT. Recognizing friends by their walk: gait perception without familiarity cues. Bull Psychon Soc. 1977:9:353–356.

[ref22] Dasgupta S, Tyler SC, Wicks J, Srinivasan R, Grossman ED. Network connectivity of the right STS in three social perception localizers. J Cogn Neurosci. 2017:29:221–234. 27991030 10.1162/jocn_a_01054

[ref33] de Gelder B, Snyder J, Greve D, Gerard G, Hadjikhani N. Fear fosters flight: a mechanism for fear contagion when perceiving emotion expressed by a whole body. Proc Natl Acad Sci USA. 2004:101:16701–16706. 15546983 10.1073/pnas.0407042101 PMC528902

[ref34] de Gelder B, Pichon S, Kret M, Grezes J. Men fear other men most: gender specific brain activations in perceiving threat from dynamic faces and bodies – an fMRI study. Nat Prec. 2010:1–1. https://www.nature.com/articles/npre.2010.4801.1. 10.3389/fpsyg.2011.00003 PMC3111446 21713131

[ref35] de Gelder B, Tamietto M, Pegna AJ, Van den Stock J. Visual imagery influences brain responses to visual stimulation in bilateral cortical blindness. Cortex Whole is Greater than the Sum of the Parts. 2015:72:15–26. 10.1016/j.cortex.2014.11.009 25571770

[ref23] De Lussanet MHE, Fadiga L, Michels L, Seitz RJ, Kleiser R, Lappe M. Interaction of visual hemifield and body view in biological motion perception. Eur J Neurosci. 2008:27:514–522. 18215244 10.1111/j.1460-9568.2007.06009.x

[ref24] Dice LR . Measures of the amount of ecologic association between species. Ecology. 1945:26:297–302.

[ref25] Dubois J, de Berker AO, Tsao DY. Single-unit recordings in the macaque face patch system reveal limitations of fMRI MVPA. J Neurosci. 2015:35:2791–2802. 25673866 10.1523/JNEUROSCI.4037-14.2015 PMC4323541

[ref26] Edelman S, Grill-Spector K, Kushnir T, Malach R. Toward direct visualization of the internal shape representation space by fMRI. Psychobiology. 1998:26:309–321.

[ref27] Forman P, Cohen JD, Fitzgerald M, Eddy WF, Noll DC. Improved assessment of significant activation in functional magnetic resonance imaging (fMRI): use of a cluster-size threshold. Magn Reson Med. 1995:33:636–647. 7596267 10.1002/mrm.1910330508

[ref28] Foster C, Zhao M, Bolkart T, Black MJ, Bartels A, Bülthoff I. The neural coding of face and body orientation in occipitotemporal cortex. NeuroImage. 2022:246:118783. 34879251 10.1016/j.neuroimage.2021.118783

[ref29] Frith U, Frith C. The social brain: allowing humans to boldly go where no other species has been. Phil Trans R Soc B. 2010:365:165–176. 20008394 10.1098/rstb.2009.0160 PMC2842701

[ref30] Fuelscher I, Caeyenberghs K, Enticott PG, Kirkovski M, Farquharson S, Lum J, Hyde C. Does fMRI repetition suppression reveal mirror neuron activity in the human brain? Insights from univariate and multivariate analysis. Eur J Neurosci. 2019:50:2877–2892. 30758079 10.1111/ejn.14370

[ref31] Gaffrey MS, Luby JL, Belden AC, Hirshberg JS, Volsch J, Barch DM. Association between depression severity and amygdala reactivity during sad face viewing in depressed preschoolers: an fMRI study. J Affect Disord. 2011:129:364–370. 20869122 10.1016/j.jad.2010.08.031 PMC3029507

[ref32] Gauthier I, Logothetis NK. Is face recognition not so unique after all? Cogn Neuropsychol. 2000:17:125–142. 20945176 10.1080/026432900380535

[ref36] Giese MA, Poggio T. Neural mechanisms for the recognition of biological movements. Nat Rev Neurosci. 2003:4:179–192. 12612631 10.1038/nrn1057

[ref37] Goebel R, Esposito F, Formisano E. Analysis of functional image analysis contest (FIAC) data with brainvoyager QX: from single-subject to cortically aligned group general linear model analysis and self-organizing group independent component analysis. Hum Brain Mapp. 2006:27:392–401. 16596654 10.1002/hbm.20249 PMC6871277

[ref38] Goldberg H, Christensen A, Flash T, Giese MA, Malach R. Brain activity correlates with emotional perception induced by dynamic avatars. NeuroImage. 2015:122:306–317. 26220746 10.1016/j.neuroimage.2015.07.056

[ref39] Gorgolewski KJ, Storkey AJ, Bastin ME, Whittle I, Pernet C. Single subject fMRI test–retest reliability metrics and confounding factors. NeuroImage. 2013:69:231–243. 23153967 10.1016/j.neuroimage.2012.10.085

[ref40] Grosbras M-H, Beaton S, Eickhoff SB. Brain regions involved in human movement perception: a quantitative voxel-based meta-analysis. Hum Brain Mapp. 2012:33:431–454. 21391275 10.1002/hbm.21222 PMC6869986

[ref41] Grossman ED, Blake R. Brain areas active during visual perception of biological motion. Neuron. 2002:35(6):1167–1175. 12354405 10.1016/s0896-6273(02)00897-8

[ref42] Grossman ED, Battelli L, Pascual-Leone A. Repetitive TMS over posterior STS disrupts perception of biological motion. Vis Res. 2005:45:2847–2853. 16039692 10.1016/j.visres.2005.05.027

[ref43] Hahn CA, O’Toole AJ. Recognizing approaching walkers: neural decoding of person familiarity in cortical areas responsive to faces, bodies, and biological motion. NeuroImage. 2017:146:859–868. 27989842 10.1016/j.neuroimage.2016.10.042

[ref44] Hall LMJ, Klimes-Dougan B, Hunt RH, Thomas KM, Houri A, Noack E, Mueller BA, Lim KO, Cullen KR. An fMRI study of emotional face processing in adolescent major depression. J Affect Disord. 2014:168:44–50. 25036008 10.1016/j.jad.2014.06.037 PMC4171128

[ref45] Haxby JV, Hoffman EA, Gobbini MI. The distributed human neural system for face perception. Trends Cogn Sci. 2000:4:223–233. 10827445 10.1016/s1364-6613(00)01482-0

[ref46] Herrington JD, Baron-Cohen S, Wheelwright SJ, Singh KD, Bullmore ET, Brammer M, Williams SCR. The role of MT+/V5 during biological motion perception in asperger syndrome: an fMRI study. Res Autism Spect Dis. 2007:1:14–27.

[ref47] Hirai M, Senju A. The two-process theory of biological motion processing. Neurosci Biobehav Rev. 2020:111:114–124. 31945392 10.1016/j.neubiorev.2020.01.010

[ref48] Howard RJ, Brammer M, Wright I, Woodruff PW, Bullmore ET, Zeki S. A direct demonstration of functional specialization within motion-related visual and auditory cortex of the human brain. Curr Biol. 1996:6:1015–1019. 8805334 10.1016/s0960-9822(02)00646-2

[ref49] Jastorff J, Orban GA. Human functional magnetic resonance imaging reveals separation and integration of shape and motion cues in biological motion processing. J Neurosci. 2009:29:7315–7329. 19494153 10.1523/JNEUROSCI.4870-08.2009 PMC6666481

[ref50] Jastorff J, Huang Y-A, Giese MA, Vandenbulcke M. Common neural correlates of emotion perception in humans. Hum Brain Mapp. 2015:36:4184–4201. 26219630 10.1002/hbm.22910 PMC6869080

[ref51] Jastorff J, De Winter F-L, Van den Stock J, Vandenberghe R, Giese MA, Vandenbulcke M. Functional dissociation between anterior temporal lobe and inferior frontal gyrus in the processing of dynamic body expressions: insights from behavioral variant frontotemporal dementia. Hum Brain Mapp. 2016:37:4472–4486. 27510944 10.1002/hbm.23322 PMC6867423

[ref52] Johansson G . Visual perception of biological motion and a model for its analysis. Percept Psychophys. 1973:14:201–211.

[ref53] Johnson KL, McKay LS, Pollick FE. He throws like a girl (but only when he’s sad): emotion affects sex-decoding of biological motion displays. Cognition. 2011:119:265–280. 21349506 10.1016/j.cognition.2011.01.016

[ref55] Kesler-West ML, Andersen AH, Smith CD, Avison MJ, Davis CE, Kryscio RJ, Blonder LX. Neural substrates of facial emotion processing using fMRI. Brain Res Cogn Brain Res. 2001:11:213–226. 11275483 10.1016/s0926-6410(00)00073-2

[ref56] Klüver M, Hecht H, Troje NF. Internal consistency predicts attractiveness in biological motion walkers. Evol Hum Behav. 2016:37:40–46.

[ref57] Kozlowski LT, Cutting JE. Recognizing the sex of a walker from a dynamic point-light display. Percept Psychophys. 1977:21:575–580.

[ref58] Kret M, Pichon S, Grezes J, De Gelder B. Men fear other men most: gender specific brain activations in perceiving threat from dynamic faces and bodies – an fMRI study. Front Psychol. 2011:2:3. 21713131 10.3389/fpsyg.2011.00003 PMC3111446

[ref59] Kriegeskorte N, Mur M, Bandettini PA. Representational similarity analysis – connecting the branches of systems neuroscience. Front Sys Neurosci. 2008:2:4. 10.3389/neuro.06.004.2008 PMC2605405 19104670

[ref1l] Lange J, Lappe M. A model of biological motion perception from configural form cues. J Neurosci. 2006:26:2894–2906. 10.1523/JNEUROSCI.4915-05.2006 PMC6673973 16540566

[ref61] Lee Y-S, Turkeltaub P, Granger R, Raizada RDS. Categorical speech processing in broca’s area: an fMRI study using multivariate pattern-based analysis. J Neurosci. 2012:32:3942–3948. 22423114 10.1523/JNEUROSCI.3814-11.2012 PMC6703443

[ref62] Liang JC, Wagner AD, Preston AR. Content representation in the human medial temporal lobe. Cereb Cortex. 2013:23:80–96. 22275474 10.1093/cercor/bhr379 PMC3513952

[ref63] Lichtensteiger J, Loenneker T, Bucher K, Martin E, Klaver P. Role of dorsal and ventral stream development in biological motion perception. Neuroreport. 2008:19:1763–1767. 18955908 10.1097/WNR.0b013e328318ede3

[ref64] Liuzzi AG, Aglinskas A, Fairhall SL. General and feature-based semantic representations in the semantic network. Sci Rep. 2020:10:1–12. 32488152 10.1038/s41598-020-65906-0 PMC7265368

[ref65] Mak AKY, Hu Z-G, Zhang JX, Xiao Z-W, Lee TMC. Neural correlates of regulation of positive and negative emotions: an fmri study. Neurosci Lett. 2009:457:101–106. 19429172 10.1016/j.neulet.2009.03.094

[ref66] McLellan TL, Wilcke JC, Johnston L, Watts R, Miles LK. Sensitivity to posed and genuine displays of happiness and sadness: a fMRI study. Neurosci Lett. 2012:531:149–154. 23123788 10.1016/j.neulet.2012.10.039

[ref67] Michels L, Kleiser R, de Lussanet MHE, Seitz RJ, Lappe M. Brain activity for peripheral biological motion in the posterior superior temporal gyrus and the fusiform gyrus: dependence on visual hemifield and view orientation. NeuroImage. 2009:45:151–159. 19063979 10.1016/j.neuroimage.2008.10.063

[ref68] Montepare JM, Zebrowitz-McArthur L. Impressions of people created by age-related qualities of their gaits. J Pers Soc Psychol. 1988:55:547–556. 3193347 10.1037//0022-3514.55.4.547

[ref69] Ng M, Ciaramitaro VM, Anstis S, Boynton GM, Fine I. Selectivity for the configural cues that identify the gender, ethnicity, and identity of faces in human cortex. Proc Natl Acad Sci USA. 2006:103:19552–19557. 17164335 10.1073/pnas.0605358104 PMC1748263

[ref70] Oosterhof NN, Connolly AC, Haxby JV. CoSMoMVPA: multi-modal multivariate pattern analysis of neuroimaging data in Matlab/GNU octave. Front Neuroinform. 2016:10:27. 27499741 10.3389/fninf.2016.00027 PMC4956688

[ref71] Osgood CE. Semantic diferential technique in the comparative study of cultures. Am Anthropol. 1964:66:171–200.

[ref72] Parkinson C, Walker TT, Memmi S, Wheatley T. Emotions are understood from biological motion across remote cultures. Emotion. 2017:17:459–477. 27819448 10.1037/emo0000194

[ref73] Peelen MV, Wiggett AJ, Downing PE. Patterns of fMRI activity dissociate overlapping functional brain areas that respond to biological motion. Neuron. 2006:49:815–822. 16543130 10.1016/j.neuron.2006.02.004

[ref74] Peuskens H, Vanrie J, Verfaillie K, Orban GA. Specificity of regions processing biological motion. Eur J Neurosci. 2005:21:2864–2875. 15926934 10.1111/j.1460-9568.2005.04106.x

[ref75] Phelps EA . Emotion and cognition: insights from studies of the human amygdala. Annu Rev Psychol. 2006:57:27–53. 16318588 10.1146/annurev.psych.56.091103.070234

[ref76] Pitcher D, Ungerleider LG. Evidence for a third visual pathway specialized for social perception. Trends Cogn Sci. 2021:25:100–110. 33334693 10.1016/j.tics.2020.11.006 PMC7811363

[ref77] Poyo Solanas M, Vaessen M, de Gelder B. Computation-based feature representation of body expressions in the human brain. Cereb Cortex. 2020:30:6376–6390. 32770200 10.1093/cercor/bhaa196

[ref78] Prochnow D, Höing B, Kleiser R, Lindenberg R, Wittsack H-J, Schäfer R, Franz M, Seitz RJ. The neural correlates of affect reading: an fMRI study on faces and gestures. Behav Brain Res. 2013:237:270–277. 22981562 10.1016/j.bbr.2012.08.050

[ref79] Riesenhuber M, Poggio T. Hierarchical models of object recognition in cortex. Nat Neurosci. 1999:2:1019–1025. 10526343 10.1038/14819

[ref80] Ross P, de Gelder B, Crabbe F, Grosbras M-H. Emotion modulation of the body-selective areas in the developing brain. Dev Cogn Neuros-neth. 2019:38:100660. 10.1016/j.dcn.2019.100660 PMC6969350 31128318

[ref81] Rousselet GA, Thorpe SJ, Fabre-Thorpe M. How parallel is visual processing in the ventral pathway? Trends Cogn Sci. 2004:8:363–370. 15335463 10.1016/j.tics.2004.06.003

[ref82] Sair HI, Yahyavi-Firouz-Abadi N, Calhoun VD, Airan RD, Agarwal S, Intrapiromkul J, Choe AS, Gujar SK, Caffo B, Lindquist MA, et al. Presurgical brain mapping of the language network in patients with brain tumors using resting-state fMRI: comparison with task fMRI. Hum Brain Mapp. 2016:37:913–923. 26663615 10.1002/hbm.23075 PMC6867315

[ref83] Salmela V, Salo E, Salmi J, Alho K. Spatiotemporal dynamics of attention networks revealed by representational similarity analysis of EEG and fMRI. Cereb Cortex. 2018:28:549–560. 27999122 10.1093/cercor/bhw389

[ref84] Sapountzis P, Schluppeck D, Bowtell R, Peirce JW. A comparison of fMRI adaptation and multivariate pattern classification analysis in visual cortex. NeuroImage. 2010:49:1632–1640. 19815081 10.1016/j.neuroimage.2009.09.066 PMC2793370

[ref85] Saunders DR, Williamson DK, Troje NF. Gaze patterns during perception of direction and gender from biological motion. J Vision. 2010:10:9–9. 10.1167/10.11.9 20884504

[ref86] Saygin AP . Point-light biological motion perception activates human premotor cortex. J Neurosci. 2004:24:6181–6188. 15240810 10.1523/JNEUROSCI.0504-04.2004 PMC6729669

[ref87] Saygin AP . Superior temporal and premotor brain areas necessary for biological motion perception. Brain. 2007:130:2452–2461. 17660183 10.1093/brain/awm162

[ref88] Sergerie K, Chochol C, Armony JL. The role of the amygdala in emotional processing: a quantitative meta-analysis of functional neuroimaging studies. Neurosci Biobehav Rev. 2008:32:811–830. 18316124 10.1016/j.neubiorev.2007.12.002

[ref89] Serre T, Oliva A, Poggio T. A feedforward architecture accounts for rapid categorization. Proc Natl Acad Sci USA. 2007:104:6424–6429. 17404214 10.1073/pnas.0700622104 PMC1847457

[ref90] Shi J, Weng X, He S, Jiang Y. Biological motion cues trigger reflexive attentional orienting. Cognition. 2010:117:348–354. 20883983 10.1016/j.cognition.2010.09.001 PMC2967601

[ref91] Sokolov AA, Zeidman P, Erb M, Ryvlin P, Friston KJ, Pavlova MA. Structural and effective brain connectivity underlying biological motion detection. Proc Natl Acad Sci USA. 2018:115:E12034–E12042. 30514816 10.1073/pnas.1812859115 PMC6305007

[ref92] Sørensen TA . A method of establishing groups of equal amplitude in plant sociology based on similarity of species and its application to analyses of the vegetation on Danish commons. Biol Skr. 1948:5:1–34.

[ref93] Taylor JC, Wiggett AJ, Downing PE. fMRI–adaptation studies of viewpoint tuning in the extrastriate and fusiform body areas. J Neurophysiol. 2010:103:1467–1477. 20032242 10.1152/jn.00637.2009

[ref94] Thompson J, Parasuraman R. Attention, biological motion, and action recognition. NeuroImage. 2012:59(1):4–13. 21640836 10.1016/j.neuroimage.2011.05.044

[ref95] Thornton MA, Mitchell JP. Consistent neural activity patterns represent personally familiar people. J Cogn Neurosci. 2017:29:1583–1594. 28557690 10.1162/jocn_a_01151

[ref96] Troje NF . Decomposing biological motion: a framework for analysis and synthesis of human gait patterns. J Vision. 2002:2:2. 10.1167/2.5.2 12678652

[ref97] Troje NF . Retrieving information from human movement patterns. Understanding Events How Humans See Represent Act Events. 2008:4:22–29.

[ref98] Turner BO, Mumford JA, Poldrack RA, Ashby FG. Spatiotemporal activity estimation for multivoxel pattern analysis with rapid event-related designs. NeuroImage. 2012:62:1429–1438. 22659443 10.1016/j.neuroimage.2012.05.057 PMC3408801

[ref54] van Kemenade BM, Muggleton N, Walsh V, Saygin AP. Effects of TMS over premotor and superior temporal cortices on biological motion perception. J Cognitive Neurosci. 2012:24:896–904. 10.1162/jocn_a_00194 22264195

[ref111] van der Zwan R, MacHatch C, Kozlowski D, Troje NF, Blanke O, Brooks A. Gender bending: auditory cues affect visual judgements of gender in biological motion displays. Exp Brain Res. 2009:198:373–382. 19396433 10.1007/s00221-009-1800-y

[ref99] Vaina LM, Solomon J, Chowdhury S, Sinha P, Belliveau JW. Functional neuroanatomy of biological motion perception in humans. Proc Natl Acad Sci USA. 2001:98:11656–11661. 11553776 10.1073/pnas.191374198 PMC58785

[ref100] Vanderhasselt M-A, Kühn S, De Raedt R. Healthy brooders employ more attentional resources when disengaging from the negative: an event-related fMRI study. Cogn Affect Behav Neurosci. 2011:11:207–216. 21373973 10.3758/s13415-011-0022-5

[ref101] Vangeneugden J, Peelen M, Tadin D, Battelli L. Double dissociation between the extrastriate body area and the posterior superior temporal sulcus during biological motion perception: converging evidence from TMS and fMRI. J Vis. 2012:12:937.

[ref102] Vangeneugden J, Peelen MV, Tadin D, Battelli L. Distinct neural mechanisms for body form and body motion discriminations. J Neurosci. 2014:34:574–585. 24403156 10.1523/JNEUROSCI.4032-13.2014 PMC6608149

[ref103] Wang L, Yang X, Shi J, Jiang Y. The feet have it: local biological motion cues trigger reflexive attentional orienting in the brain. NeuroImage. 2014:84:217–224. 23994124 10.1016/j.neuroimage.2013.08.041

[ref104] Wang L, Wang Y, Xu Q, Liu D, Ji H, Yu Y, Hu Z, Yuan P, Jiang Y. Heritability of reflexive social attention triggered by eye gaze and walking direction: common and unique genetic underpinnings. Psychol Med. 2020:50:475–483. 30829191 10.1017/S003329171900031X

[ref105] Weiner KS, Grill-Spector K. Not one extrastriate body area: using anatomical landmarks, hMT+, and visual field maps to parcellate limb-selective activations in human lateral occipitotemporal cortex. NeuroImage. 2011:56:2183–2199. 21439386 10.1016/j.neuroimage.2011.03.041 PMC3138128

[ref106] Westhoff C, Troje NF. Kinematic cues for person identification from biological motion. Percept Psychophys. 2007:69:241–253. 17557594 10.3758/bf03193746

[ref107] Xia M, Wang J, Yong H, Peter C. BrainNet viewer: a network visualization tool for human brain connectomics. PLoS One. 2013:8:e68910. 23861951 10.1371/journal.pone.0068910 PMC3701683

[ref108] Yang X, Cai P, Jiang Y. Effects of walker gender and observer gender on biological motion walking direction discrimination: gender modulates biological motion perception. PsyCh J. 2014:3:169–176. 26271936 10.1002/pchj.53

[ref109] Yoshino A, Okamoto Y, Onoda K, Yoshimura S, Kunisato Y, Demoto Y, Okada G, Yamawaki S. Sadness enhances the experience of pain via neural activation in the anterior cingulate cortex and amygdala: an fMRI study. NeuroImage. 2010:50:1194–1201. 19969094 10.1016/j.neuroimage.2009.11.079

[ref110] Yovel G, O’Toole AJ. Recognizing people in motion. Trends Cogn Sci. 2016:20:383–395. 27016844 10.1016/j.tics.2016.02.005

