# Supplementary material for: Distributed and hierarchical neural encoding of multidimensional biological motion attributes in the human brain
Source: Cereb Cortex. 2023 Apr 28;33(13):8510–22. doi: 10.1093/cercor/bhad136 (PMC10786095; doi:10.1093/cercor/bhad136)
Supplement: SupplementaryMaterial_bhad136 [file supplementarymaterial_bhad136.doc]

Supplementary material for

**Distributed and hierarchical neural encoding of** **multi-dimensional biological motion attributes in the human brain**

**Ruidi Wang^1,2,3^, Xiqian Lu^1,2,3,*^, Yi Jiang^1,2,3,*^**

^1^State Key Laboratory of Brain and Cognitive Science, CAS Center for Excellence in Brain Science and Intelligence Technology, Institute of Psychology, Chinese Academy of Sciences, 16 Lincui Road, Beijing 100101, China

^2^Department of Psychology, University of Chinese Academy of Sciences, 19A Yuquan Road, Beijing 100049, China

^3^Chinese Institute for Brain Research, 26 Science Park Road, Beijing 102206, China

*Corresponding author at: Institute of Psychology, Chinese Academy of Sciences, 16 Lincui Road, Beijing 100101, China

*E-mail address:* Xiqian Lu ([luxq@psych.ac.cn](mailto:luxq@psych.ac.cn)) and Yi Jiang [(yijiang@psych.ac.cn)](mailto:(yijiang@psych.ac.cn))

**Behavioral experiment.**

**Table S1 shows each participant’s mean scores in discriminating the three BM attributes.**

| **Table S1. Behavioral results for BM attributes discrimination task** | | | | | | |
| --- | --- | --- | --- | --- | --- | --- |
| Participant No. | Facing direction | | Gender | | Emotional state | |
|  | Left | Right | Female | Male | Sad | Happy |
| 01 | 1.00 | 7.00 | 1.63 | 6.00 | 3.38 | 6.00 |
| 02 | 1.00 | 7.00 | 1.00 | 6.00 | 2.50 | 6.94 |
| 03 | 1.00 | 6.88 | 1.81 | 6.40 | 1.31 | 6.13 |
| 04 | 1.44 | 6.63 | 2.13 | 5.92 | 3.38 | 5.21 |
| 05 | 1.00 | 7.00 | 1.00 | 7.00 | 1.25 | 7.00 |
| 06 | 1.00 | 7.00 | 1.38 | 6.06 | 3.06 | 5.88 |
| 07 | 1.00 | 7.00 | 1.00 | 6.94 | 1.00 | 6.94 |
| 08 | 1.00 | 6.94 | 1.08 | 7.00 | 1.00 | 6.88 |
| 09 | 1.06 | 7.00 | 2.35 | 6.19 | 2.67 | 5.92 |
| 10 | 1.00 | 7.00 | 2.06 | 5.75 | 2.56 | 5.75 |
| 11 | 1.00 | 7.00 | 1.46 | 6.19 | 1.17 | 6.88 |
| 12 | 1.00 | 7.00 | 1.75 | 6.00 | 2.63 | 6.25 |
| 13 | 1.13 | 7.00 | 1.38 | 6.06 | 2.13 | 6.44 |
| 14 | 1.00 | 7.00 | 1.00 | 7.00 | 1.33 | 6.94 |
| 15 | 1.00 | 7.00 | 1.94 | 5.71 | 2.90 | 6.00 |
| 16 | 1.00 | 6.81 | 2.06 | 6.06 | 2.81 | 5.54 |
| 17 | 1.79 | 5.94 | 1.56 | 6.25 | 2.94 | 6.00 |
| 18 | 1.19 | 6.81 | 1.06 | 6.44 | 2.38 | 6.17 |
| 19 | 1.19 | 6.94 | 1.63 | 6.13 | 2.00 | 6.00 |
| 20 | 1.00 | 6.94 | 2.73 | 5.52 | 3.04 | 5.56 |

**Forward- or backward-walking did not affect the perception of multi-dimensional biological motion (BM) attributes.** In the fMRI experiment, the stimuli were presented in either a forward-walking or backward-walking manner. To further investigate whether the different walking directions influence the recognition of the three BM attributes, we conducted a behavioral experiment on a new group of 21 participants (13 female, aged 23 to 30 years). Participants were required to judge an attribute of the BM stimulus on a three-point scale when the walking direction of the BM stimulus was forward-walking or backward-walking. Each of the 8 BM sequences with forward-walking or backward-walking was estimated three times regarding its facing direction, gender, and emotional state, leading to a total of 144 trials. Specifically, in a trial, if the question is “what is the facing direction of the person”, the option “left” will be given 1, “not sure” will be given 2, and “right” will be given 3; if the question is “what is the gender of the person”, and the option “female” will be given 1, “not sure” was given 2, and “male” will be given 3; if the question is “what is the emotional state of the person”, and the option “sad” will be given 1, “not sure” was given 2, and “happy” will be given 3. Results showed that the recognition of facing direction, gender, and emotion were not significantly influenced by different walking directions (Table S2).

| **Table S2. BM attributes recognition results for forward-walking and backward-walking sequences** | | | |
| --- | --- | --- | --- |
|  |  | Mean±SD | |
|  |  | Forward | Backward |
| Facing direction | Left | 1.01±0.03 | 1.03±0.08 |
|  | Right | 3±0.02 | 2.94±0.16 |
| Gender | Female | 1.14±0.25 | 1.30±0.36 |
|  | Male | 2.81±0.30 | 2.73±0.33 |
| Emotional state | Sad | 1.40±0.24 | 1.28±0.20 |
|  | Happy | 2.91±0.15 | 2.91±0.14 |

**The V1 representation of biological motion attributes.** The current study focused on the brain representations of distinct BM attributes. In order to exclude brain activity simply caused by the early visual processing of the stimuli, we developed a feature model by using the HMAX model. The HMAX model simulates the object recognition process in early visual cortex nerve cells (Serre et al. 2007). The C1 layer of the HMAX model is widely used to simulate the V1 representation of the silence pictures feature (Kriegeskorte et al. 2008; Connolly et al. 2012; Alfred et al. 2018; Tucciarelli et al. 2019; Grootswagers et al. 2020; Bayer et al. 2021). We calculated the HMAX-C1 response using every frame of the BM stimuli, and then obtained the mean response in each stimulus’ sequence by using the mean response of all frames. To prove our hypothesis, we calculated the brain representation of V1 representation similarity matrix (RDM) by representation similarity analysis (RSA). Results revealed that the HMAX-C1 layer was represented in the early visual areas (Fig. S1).


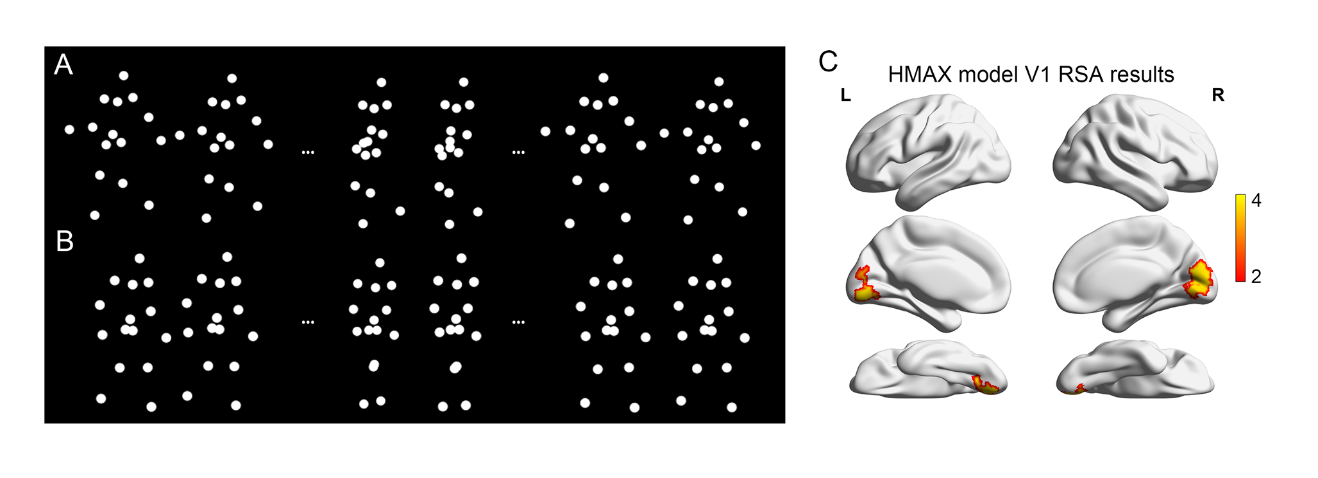


**Fig. S1. V1 RSA calculation materials and results.** The female point-light stimulus, facing to the left, with a happy mood (A) was split into 30 frames, every frame was put into the HMAX-model and extracted the C1 layer’s response. All responses of the 30 frames were then averaged. (B) A male point-light stimulus, facing to the right, with a sad mood. All other point-light stimuli used the same methods to get the C1 layer’s responses. Then we derived the V1 RDM using 1 - Pearson correlation as a measure of dissimilarity. Using the V1 RDM, we obtained the V1 RSA results (C). The results were corrected by Monte Carlo simulations (*p* = .001 at the initial voxel wise, and 5000 iterations).

**Multiple regression RSA with the three theory RDMs but without the V1 RDM as predictors.** Results showed similar cortical maps with the multiple regression RSA (Fig. 2 of the article) for each attribute except the visual network.

**
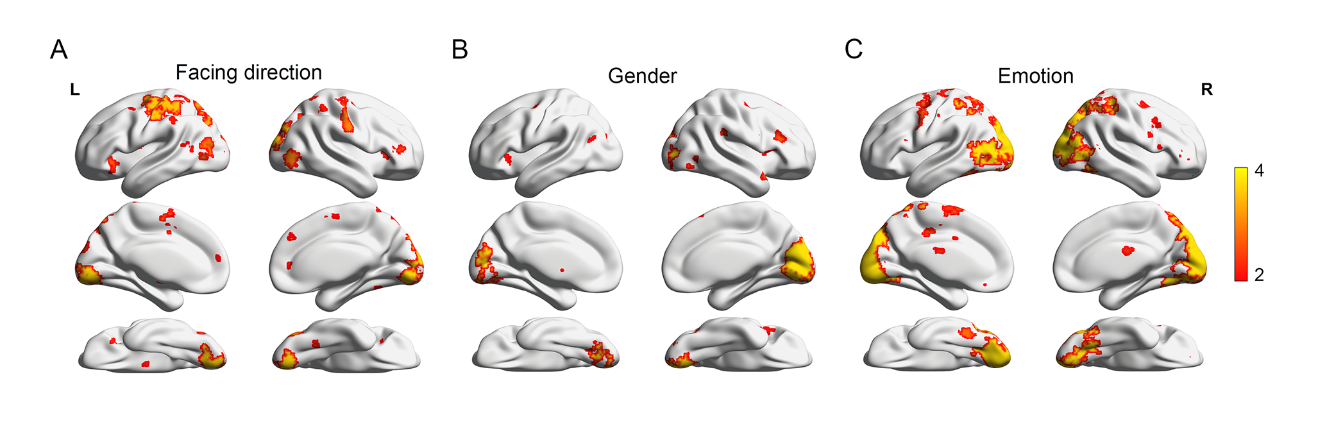
Fig. S2. Multiple regression RSA results with searchlight method.** (A) The facing direction RSA results calculated by facing direction RDM with gender and emotion RDMs disrupted. (B) The gender RSA results calculated by gender RDM with facing direction and emotion RDMs disrupted. (C) The emotion RSA results calculated by emotion RDM with facing direction and gender RDMs disrupted. The results were corrected by Monte Carlo simulations (*p* = .001 at the initial voxel wise, and 5000 iterations).


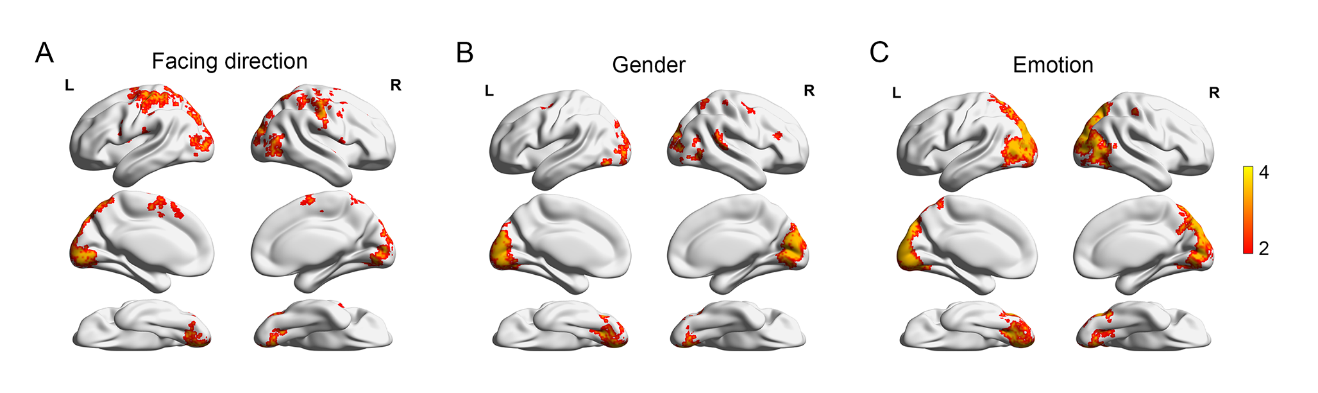


**Fig. S3. Results of multi-voxel pattern analysis (MVPA) with searchlight method.** (A) The MVPA results of the facing direction attribute. (B) The MVPA results of the gender attribute. (C) The MVPA results of the emotion attribute. The results were corrected by Monte Carlo simulations (*p* = .001 at the initial voxel wise, and 5000 iterations).

**Permutation test on MVPA results.** We also evaluated the significance of MVPA results by using permutation testing. For each ROI, each of the three classification analyses was repeated 1000 times with the condition labels randomly assigned to generate a null distribution of mean classification accuracies expected by chance. From the distribution of the classification accuracy values resulting from these randomly resampled analyses, we obtained the 95% confidence interval for chance performance. Finally, we assessed significance using the threshold of *p* < .05. The results showed the similar brain networks to those obtained when comparing against chance level (50%) for the three attributes. For facing direction, the bilateral lingual gyri (BA 17), left MOG (BA 37), right SOG (BA 18), right MTG (BA 19), bilateral FG (BA 18), bilateral SPL (BA 7), bilateral postcentral gyri (BA 3), left precentral gyrus (BA 4), left SMA (BA 6), right insula (BA 13), and left IFG (BA 47) were significant (Fig. S4A). For gender, right lingual gyrus (BA 17), left FG (BA 37), right ITG (BA 37), right pSTS (BA 22), right SMA (BA 6), right insula (BA 13), right IFG (BA 47), and right MFG (BA 6) were significant (Fig. S4B). For emotional state, bilateral lingual gyri (BA 17, 18), bilateral IOG (BA 18), bilateral MOG (BA 19), bilateral SOG (BA 7, 18, 19), bilateral FG (BA 19), bilateral ITG (BA 37), bilateral MTG (BA 39), bilateral IPL (BA 40), bilateral SPL (BA 7), bilateral postcentral gyri (BA 3), right precentral gyrus (BA 6), and left SMA (BA 6) were significant (Fig. S4C).


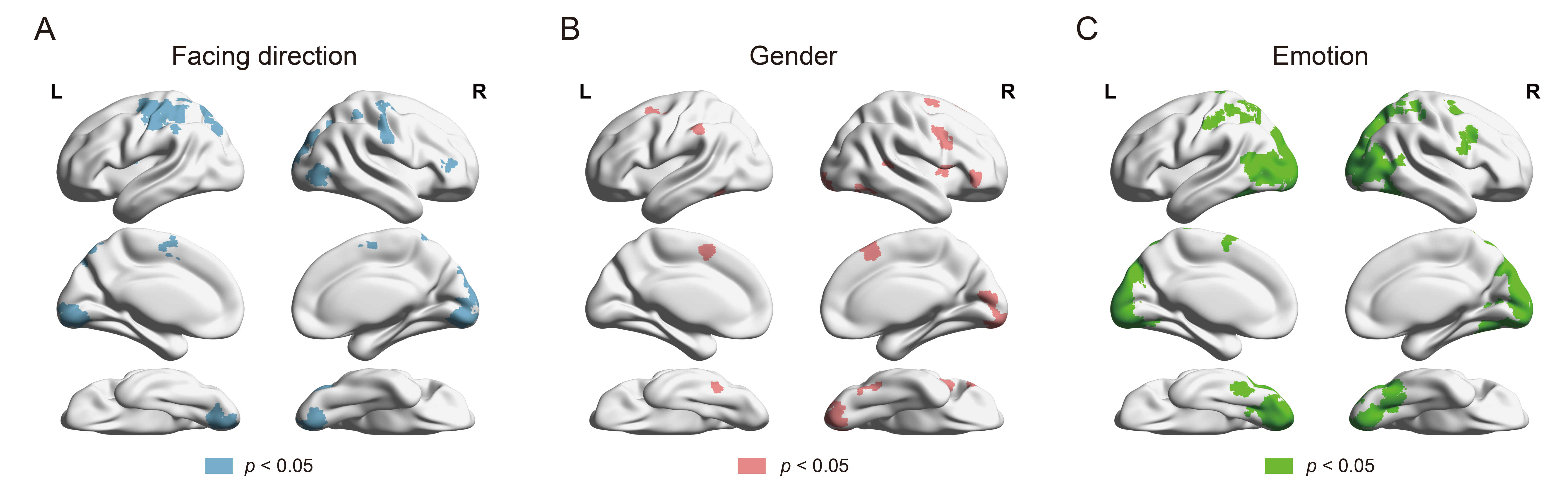


**Fig. S4. ROI-based MVPA results corrected by permutation test (*p* < .05).** ROI were defined by the significant clusters in RSA of facing direction (A), gender (B), and emotional state (C).

**References**

Alfred KL, Connolly AC, Kraemer DJM. 2018. Putting the pieces together: Generating a novel representational space through deductive reasoning. NeuroImage. 183:99–111.

Bayer M, Berhe O, Dziobek I, Johnstone T. 2021. Rapid Neural Representations of Personally Relevant Faces. Cereb Cortex N Y N 1991. 31:4699–4708.

Connolly AC, Guntupalli JS, Gors J, Hanke M, Halchenko YO, Wu Y-C, Abdi H, Haxby JV. 2012. The Representation of Biological Classes in the Human Brain. J Neurosci. 32:2608–2618.

Grootswagers T, Kennedy BL, Most SB, Carlson TA. 2020. Neural signatures of dynamic emotion constructs in the human brain. Neuropsychologia. 145:106535.

Kriegeskorte N, Mur M, Bandettini PA. 2008. Representational similarity analysis – connecting the branches of systems neuroscience. Front Syst Neurosci. 2:4.

Serre T, Oliva A, Poggio T. 2007. A feedforward architecture accounts for rapid categorization. Proc Natl Acad Sci. 104:6424–6429.

Tucciarelli R, Wurm M, Baccolo E, Lingnau A. 2019. The representational space of observed actions. eLife. 8:e47686.
